# The Mechanisms of Improving IVF Outcomes of Liu-Wei-Di-Huang Pill Acting on DOR Patients

**DOI:** 10.1155/2020/5183017

**Published:** 2020-10-31

**Authors:** Jimei Xiao, Jingyan Song, Yuanhong Sa, Lihua Yuan, Jiayin Guo, Zhengao Sun

**Affiliations:** ^1^The First Clinical College, Shandong University of Traditional Chinese Medicine, Jinan 250014, China; ^2^Guangdong Provincial Key Laboratory of New Drug Screening, School of Pharmaceutical Sciences, Southern Medical University, Guangzhou 510515, China; ^3^Reproductive and Genetic Center of Integrated Traditional and Western Medicine, The Affiliated Hospital of Shandong University of Traditional Chinese Medicine, Jinan 250011, China

## Abstract

Diminished ovarian reserve (DOR) is the weakening of ovarian oocyte production and quality. It will further become premature ovarian failure without timely cure. However, disease pathology and diagnostic markers are still incompletely understood. Liu-Wei-Di-Huang (LWDH) pill, a traditional Chinese medicine formula, is commonly used in the treatment of DOR in China. To explore the mechanism of the effect of LWDH on in vitro fertilization (IVF) outcomes in patients with DOR, a pseudotargeted metabolomics study combined with multivariate data processing strategy was carried out. A liquid chromatography tandem mass spectrometry-based metabolomics approach was applied to characterize metabolic biomarker candidates. Multiple pattern recognition was used to determine groups and confirm important variables. A total of 21 potential biomarkers were characterized, and related metabolic pathways were identified. The study displayed that the established pseudotargeted metabolomics strategy is a powerful approach for investigating the mechanism of DOR and LWDH. In addition, the approach may highlight biomarkers and metabolic pathways and can capture subtle metabolite changes from headache, which may lead to an improved mechanism understanding of DOR diseases and LWDH treatment.

## 1. Introduction

Diminished ovarian reserve (DOR) is often found in the process of long-term infertility seeking assisted pregnancy, which affects female sex hormone levels and fertility [1]. Previous studies have found that the incidence of DOR is gradually increasing and showing a younger trend [2]. In assisted reproductive technology, ovarian reserve has become an important factor affecting the outcome of assisted reproductive technology (ART). Liu-Wei-Di-Huang (LWDH) pill is a traditional Chinese medicine (TCM) formula for the treatment of DOR, with high efficiency and low toxicity in China [3]. It provides a good clinical effect in restoring menstrual cycle, protecting ovarian function in advance, and improving ART outcomes [4]. Xia and Wang improved ovarian function in patients with DOR by reestablishing yin-yang balance of heart-kidney-uterus axis and restoring normal reproductive endocrine using LWDH [5]. However, the specific mechanism is not yet clear.

Follicular fluid is a product of secretion of granulosa cells and testicular cells through the serum follicular barrier to provide nutrients for oocyte development [6]. The change in follicular fluid microenvironment will affect the occurrence and quality of oocytes [7]. At present, metabolomics as a powerful analytical strategy is applied in many fields such as biomarker discovery and TCM study [8, 9], which could provide the actual representation of the system [10]. Liquid chromatography tandem mass spectrometry (LC-MS) has become a preferred strategy in metabolomics study [11]. LC-MS mainly includes triple quadrupole mass spectrometry (QqQ), triple quadrupole-linear ion trap mass spectrometry (QTRAP), quadrupole-time of flight mass spectrometry (QTOF), and orbitrap mass spectrometry. QqQ or QTRAP could provide the analytical sequence stability and repeatability [12]. Despite orbitrap has a higher resolution rate, the QTOF analyzer possesses a much higher scanning rate, which is more suitable in the complex system and biofluids than orbitrap [13]. Therefore, QTOF- and QqQ-based pseudotargeted metabolomics method [14] was developed to characterize the metabolic biomarkers on SOR. Pseudotargeted metabolomics, taking into account the advantages of nontargeting metabolomics and targeting metabolomics, overcomes their respective shortcomings [15]. It could provide high-quality and rich-information data for metabolomics study [16]. A pattern recognition approach was carried out to estimate the changes in metabolite levels in follicular fluid and to identify 21 potential biomarkers of SOR. The integrated metabolomics study demonstrated that LWDH pill could regulate differently for different potential biomarkers. It may have more effective targets, with more comprehensive actions on the improvement of DOR. The mechanism of LWDH in treating DOR of kidney-yin deficiency was explored to provide scientific basis for clinical treatment.

## 2. Materials and Methods

### 2.1. Materials

HPLC-grade methanol, acetonitrile, water, ammonium acetate, and formic acid were all supplied by Fisher Scientific (Fair Lawn, NJ, USA). The internal standards of chloramphenicol and clenbuterol were from Sigma-Aldrich (St. Louis, MO, USA). In our study, 3.2 kg of prepared radix rehmanniae, 1.6 kg of prepared rhizoma dioscoreae, 1.6 kg of prepared fructus corni, 1.2 kg of prepared tuckahoe, 1.2 kg of prepared rhizoma alismatis, and 1.2 kg of prepared tree peony bark were crushed by pulverizer. Then, the refine honey was added to the herbal powder at a ratio of 3 to 10. They were kneaded for half an hour and prepared into LWDH pills with a mold. LWDH pills were obtained from the Affiliated Hospital of Shandong University of Traditional Chinese Medicine (Jinan, China) and authenticated by Dr. Sun (Shandong University of Traditional Chinese Medicine, Jinan, China).

### 2.2. Subjects

For the pseudometabolomics analysis, the MetSizeR approach for sample size estimation was used to estimate a total sample size of 84 subjects using the following assumptions: 954 follicular fluid metabolites, a target false detection rate of 5%, and an expected proportion of significant metabolites of 20% [17]. Subjects needed (*n* = 84) were recruited, and their follicular fluid was collected at the Affiliated Hospital of Shandong University of Traditional Chinese Medicine, from May 2018 to February 2019. For the purpose of this study, the subjects were divided into the pretreatment group (*n* = 28), the posttreatment group (*n* = 28), and the control group (*n* = 28). The diagnosis of DOR is done via expert consensus on hormone replacement therapy for premature ovarian insufficiency [18]. Subjects in the treatment group orally took one LWDH pill each time, twice a day, until the day of hCG trigger. The study was approved by the Health Authorities and Ethics Committees of the Affiliated Hospital of Shandong University of Traditional Chinese Medicine. All subjects signed the informed consent prior to being included in the study.

### 2.3. Inclusion and Exclusion Criteria

Inclusion criteria of the DOR patients are as follows: (1) kidney-yin deficiency of traditional Chinese medicine; (2) 20–40-year-old; (3) 10 IU/ml ＜ FSH ＜ 25 IU/ml; (4) patients of the control group who had a history of induced abortions of a normal pregnancy.

Inclusion criteria of the control group are as follows: (1) infertility due to male factor; (2) 20–40-year-old.

Exclusion criteria are as follows: (1) congenital genital dysplasia; (2) cerebrovascular and cardiovascular; (3) hyperprolactinemia and polycystic ovary syndrome; (4) fail to receive treatment as required due to personal and other reasons; (5) poor treatment effect.

### 2.4. Collection and Preparation of Follicular Fluid

Follicular fluid samples (approximately 1.0 mL) were collected according to the previous protocol [19]. Follicular fluid samples of 100 *μ*L were mixed with 300 *μ*L of methanol containing 500 ng/mL of internal standards. The mixture was vortexed for 2 min and then centrifuged at 13000 ×*g* for 20 min, at 4°C. The supernatant was then transferred to an autosampler plate for analysis.

### 2.5. Mass Spectrometry Analysis

The separation was performed using a SCIEX ExionLC AD ultraperformance liquid chromatography system (SCIEX, CA, USA), while the chromatographic separation was performed on a Waters HILIC (100 mm *∗* 2.1 mm, 1.7 *μ*m) column (Waters, Milford, MA, USA) at 40°C. Analysis was completed with a gradient elution of 0.1% formic acid in acetonitrile (*A*) and 10 mM ammonium acetate in water (B) within 10.0 min. The gradient program was 5% B at 0.0–0.3 min, 95% BA at 5.0–8.0 min, and 5% B at 8.1–10.0 min at a flow rate of 0.4 mL/min with a sample injection volume of 10.0 *μ*L. All the samples were kept at 4°C during the analysis.

The MS system was performed using a SCIEX QTRAP5500 (SCIEX, Redwood City, CA, USA). The multireaction monitor (MRM) and enhanced production (EPI) data were acquired in positive/negative electrospray ionization mode with dynamic background subtraction (DBS). The MRM transitions were extracted from the follicular fluid MS/MS spectra, which were acquired from TripleTOF5600 (SCIEX, Redwood City, CA, USA). Source parameters were defined as follows: temperature, 550°C; ion spray voltage, 5500 V (positive)/-4500 V (negative); nebulizer gas (gas 1), 60 psi; heater gas (gas 2), 60 psi; curtain gas, 30 psi; and declustering potential, 60 V. For IDA, any MRM survey scan peak exceeding 150 cps was selected for dependent scan. 5 candidate ions were allowed per cycle. The collision energy was set to 35 V (positive)/-35 V (negative) with a collision energy spread of 15 V. All operations and data acquisition were controlled by the Analyst 1.6 software (SCIEX, Redwood City, CA, USA).

### 2.6. Data Processing and Multivariate Data Analysis

The LC-MS data files were imported into MQ software (SCIEX, Redwood City, CA, USA). The chromatographic peaks were extracted, respectively, while Pareto scaling and multivariate analyses of log transformation were applied to the data processing before LPS-DA was performed to give the contribution of the metabolites by VIP score. All the compounds with significance threshold satisfying corrected *p* value cutoff 0.05 in one-way ANOVA and VIP score > 1 were considered as potential biomarkers. The software used for other statistical analysis was MetaboAnalyst software 4.0 (Xia Lab at McGill University, Montreal, QC, Canada).

### 2.7. Metabolic Pathway

OS software (SCIEX, Redwood City, CA, USA) was used to help confirm the potential differential metabolites by analyzing with the fragmentation patterns, chromatographic retention characteristics, and comparing the mass-spectra with the authentic standards. The fragmentation of the potential metabolites was matched with the metabolites from online databases HMDB (http://www.hmdb.ca).

Pathway analysis using the KEGG pathway database was performed by MetaboAnalyst software for the identified potential metabolites with significant changes in identifying the top DOR-correlated metabolic pathways. Meanwhile, pathway topology analysis was used to generate the impact value of the relative metabolic pathways using a relative-betweenness centrality test.

## 3. Results

### 3.1. Clinical Background

There was no significant difference in age, infertility years, body mass index, basic LH, basic E2, among basic *p* (*p* > 0.05) among the posttreatment group, the pretreatment group, and the control group. The results are shown in Table 1 for details.

### 3.2. Syndrome Integral

The total syndrome integral of DOR patients decreased significantly after treatment using LWDH pill (*p* < 0.001) (seen in Table 2). The syndrome including YaoXiSuanRuan, WuXinFanRe, KouGanYanZao, dizziness, emaciation, and forgetfulness was significantly improved after treatment (*p* < 0.05). It is suggested that LWDH pill can improve the syndrome of kidney-yin deficiency of DOR patients. The results are shown in Table 3.

### 3.3. Clinical Results

There was no significant difference in hCG daily endocrine between the pretreatment and posttreatment (*p* > 0.05). The number of oocytes (<14 mm follicles or ≥ 14 mm follicles) was statistically different (*p* < 0.05) during the day of hCG between pretreatment and posttreatment, which indicated that LWDH pill could improve the ovarian reserve of DOR patients and effectively stimulate the growth of follicles. The results showed that there were significant differences between pretreatment and posttreatment in the number of transplantable embryos and IVF fertilization rate (*p* < 0.05). However, there was no significant difference in the level of E2, the level of P, the number of high-quality embryos, the oocyte acquisition rate, and the clinical pregnancy rate (*p* > 0.05). The detail information is shown in Table 4.

### 3.4. Multivariate Statistical Analysis of Metabolite Profiling

**Figure 1:**
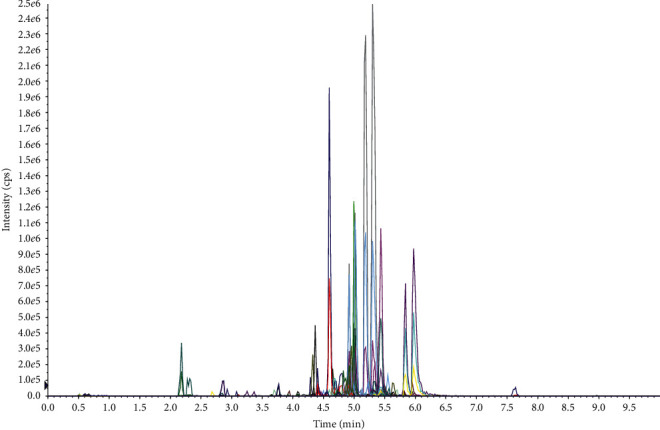
The typical chromatograms of follicular fluid samples.

**Figure 2:**
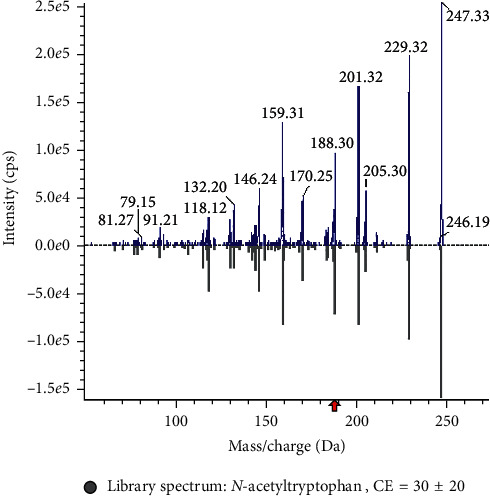
The comparison spectrum of *N*-acetyltryptophan between the experimental and reference MS/MS spectrum.

**Figure 3:**
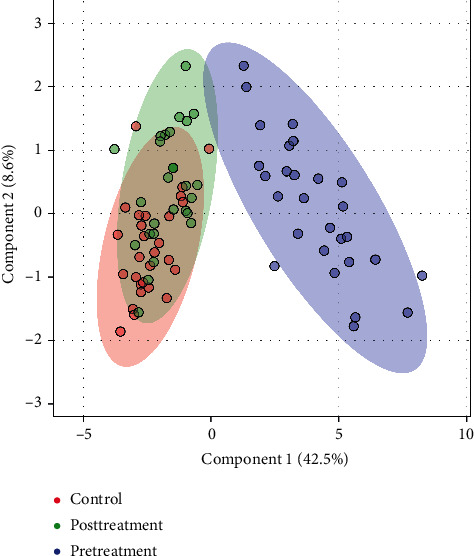
The partial least-squares discriminant analysis recognition based on the follicular fluid metabolomic profiling.

**Figure 4:**
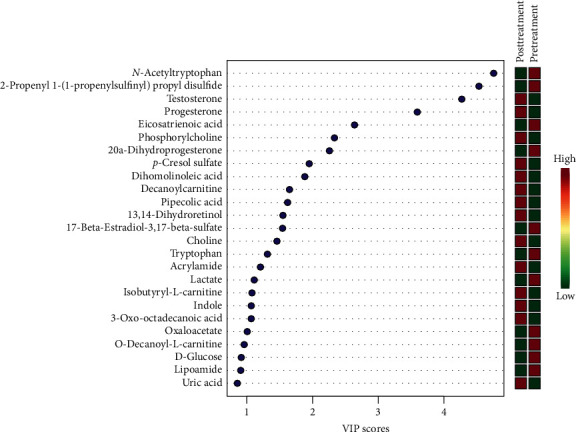
The VIP scores of each potential biomarker to the discrimination between the pretreatment group and the posttreatment group.

**Figure 5:**
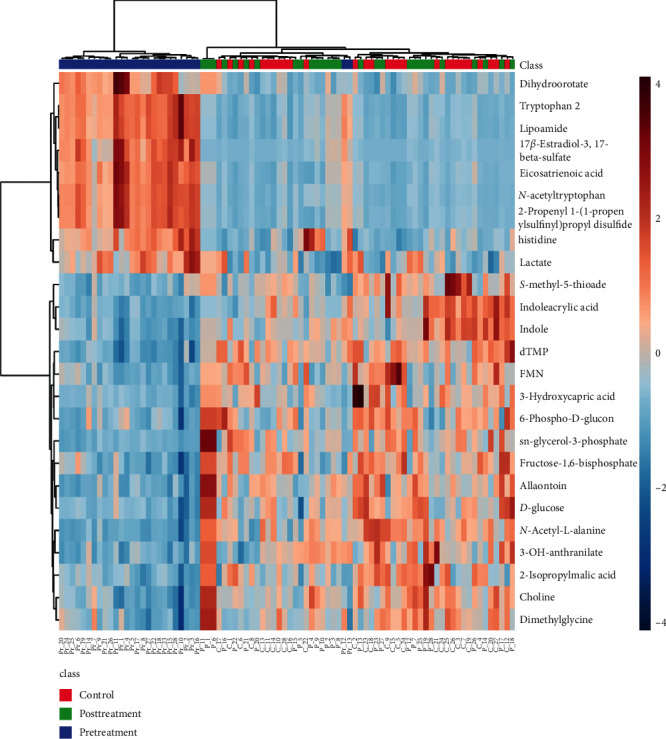
The hierarchical clustering heatmap of the potential biomarkers.

A large-scale multireaction monitor (MRM) was applied in the LC-MS detection. Typical chromatograms of follicular fluid are shown in Figure 1. In order to eliminate the complex matrix interference of follicular fluid and confirm them, their MS/MS spectrum was compared with the reference MS/MS spectrum. For example, the comparison spectrum of *N*-acetyltryptophan is shown in Figure 2. Chromatographic peaks were then carried out for alignment and normalization, followed by multivariate statistical analysis. Partial least-squares discriminant analysis (LPS-DA), a supervised multivariate analysis comparing with principal component analysis (PCA), was employed for metabolomics study to differentiate among the groups. As shown in the PLS-DA score plots, follicular fluid samples from the control group, the pretreatment group, and the posttreatment group were well separated into three categories (Figure 3), suggesting that metabolic perturbation significantly occurred in DOR patients as well as the LWDH pill treatment. All significantly differentiated metabolites satisfying corrected *p* value cutoff 0.05 in one-way ANOVA were listed. A total of 21 potential metabolic biomarkers were found after follicular fluid metabolomics screening. The results indicated that the level of testosterone, progesterone, phosphorylcholine, *p*-cresol sulfate, dihomolinoleic acid, decanoylcarnitine, pipecolic acid, 13,14-dihydroretinol, choline, acrylamide, isobutyryl-L-carnitine, indole, and 3-oxo-octadecanoic acid in follicular fluid were downregulated in DOR patients, and inversely, the level of *N*-acetyltryptophan, 2-propenyl 1-(1-propenylsulfinyl)propyl disulfide, eicosatrienoic acid, 20*α*-dihydroprogesterone, 17-beta-estradiol-3,17-beta-sulfate, tryptophan, lactate, oxaloacetate, and O-decanoyl-L-carnitine upregulated. After the treatment of LWDH, the level of all 21 biomarkers in follicular fluid significantly backregulated comparing with the pretreatment group. The contribution of each potential biomarker to the discrimination between the two groups was ranked as VIP scores shown in Figure 4. The heatmap (Figure 5) using MetaboAnalyst 4.0 demonstrated different distribution patterns of totally 21 potential biomarkers among the four groups, and moreover, the results of hierarchical cluster analysis (HCA) provided a distinct visualization of the groups [20].

### 3.5. Pathway Analysis and Metabolic Network

**Figure 6:**
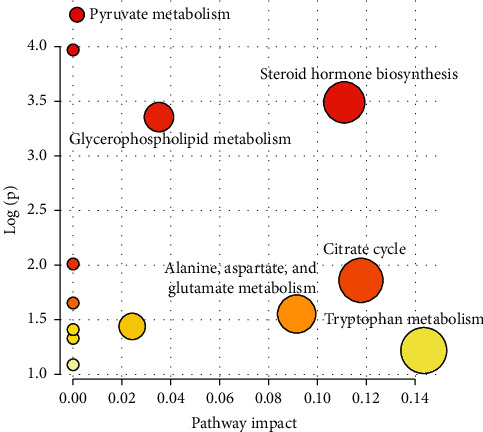
The metabolic pathways related to DOR, as analyzed by MetaboAnalyst.

The metabolic pathways related to the identified potential biomarkers were estimated by pathway topology analysis using MetaboAnalyst based on the KEGG reference pathways, in order to find possible metabolic pathways that have great impact on DOR. A figure of metabolic pathway impact which demonstrated the pathway impact values is shown in Figure 6. The results indicated that steroid hormone biosynthesis, glycerophospholipid metabolism, citrate cycle, alanine, aspartate and glutamate metabolism, and tryptophan metabolism would be considered closely related to DOR (impact > 0.01). Consequently, based on the level of the backregulation on biomarkers after the LWDH treatment, these metabolic pathways were presumed as the pathways related to the treatment of LWDH for DOR.

## 4. Discussion

The clinical manifestations of DOR were including low menstrual volume, irregular menstruation, and infertility. Patients with DOR often have symptoms of deficiency of yin and essence, such as palpitation, sweating, irritability, insomnia, and forgetfulness. As the most basic essence of human life, the kidney stores essence. When it is sufficient, women can be gestated by menstruation. With the increase in age, kidney essence gradually decreased, and reproductive function declined. LWDH pill could fill lean marrow and nourish yin and kidney. The integral of the posttreatment group was significantly lower than that of the pretreatment group, seen in Table 2. In clinical, it was found that there were significant differences in the number of < 14 mm follicles and ≥ 14 mm follicles between the posttreatment group and the pretreatment group. It shows that LWDH pill can improve the clinical symptoms of DOR patients.

Choline is essential for acetylcholine biosynthesis. Acetylcholine is a key neurotransmitter for neuron differentiation and maturation. A large intake of choline during pregnancy is beneficial to the development of embryo and the cognitive function of offspring [21, 22]. During pregnancy, there is a high demand for choline, and large intake of choline does not increase the content of choline in urine [23, 24]. Choline is also the main dietary source of methyl and affects DNA methylation as a methyl donor, while DNA methylation is related to apoptosis [25, 26]. Linoleic acid can be metabolized to other biological activities, such as arachidonic acid. It has been found that the concentration of linoleic acid in the dominant follicle is high, and the concentration of arachidonic acid is low [27]. Linolenic acid has a favorable effect on the maturation of goat oocytes in vitro, which can promote the development of embryos and reduce the expression of apoptosis genes [28]. 3-Oxo-octadecanoic acid is the intermediate of fatty acid biosynthesis. It may affect the oocyte quality by affecting fatty acid metabolism. After intervention of traditional Chinese medicine indicates, the content of 3-oxo-octadecanoic acid was upregulated near into the normal level. Carnitine is necessary for energy metabolism [29]. It will make active fatty acids into mitochondria, where they are oxidized and broken down. Carnitine can protect the continuous growth of oocytes and embryos and improve the quality of oocytes and the potential of embryo development [30] and pregnancy rate [31]. It promotes the maturation of porcine oocytes and the development of parthenogenetic embryos by accelerating nuclear maturation and preventing oxidative damage and apoptosis [32]. In vitro carnitine can improve the outcome of embryos in the culture medium for oocyte maturation and embryo growth [33]. Fathi et al. found that the rate of cleavage and embryo development was faster when L-carnitine was added during in vitro maturation (IVM), and the development rate of morula and blastocyst stages was higher [34]. You et al. found that carnitine during IVM of oocytes improved the developmental ability of SCNT embryos [35]. It may be that the increase in GSH synthesis in the recipient oocytes reduces ROS level and stimulates nuclear reprogramming by increasing the expression of Pou5f1 and transcription factors. Some studies have shown that carnitine has a protective effect on the nephrotoxicity, and the possible mechanism is to inhibit the production of reactive oxygen species, lipid peroxidation, matrix remodeling, and apoptosis, as well as anti-inflammatory [36].

Pyruvic acid is a kind of weak organic acid. It is the intermediate substance of Chinese medicine for the synthesis and decomposition of many substances, including oxidation metabolism, gluconeogenesis pathway, tricarboxylic acid cycle, and lipid synthesis. Lactic acid is the metabolite of glycolysis, which is closely related to energy metabolism, lipid metabolism, protein metabolism, and glucose metabolism. It was found that glucose and pyruvate affect the cytoplasmic maturation of porcine oocytes through glycolysis. Pyruvate can improve ROS, GSH, ATP, and early embryonic development of MII oocytes and promote the cytoplasmic maturation of pigs by providing energy and reducing oxidative stress [37, 38]. In our study, after the treatment of LWDH pill, lactate was downregulated near into the normal level in patients with DOR, which may have an impact on the quality of oocytes.

Testosterone is a kind of anabolic steroid hormone. It plays an important role in ovarian response through androgen receptor (AR) pathway and non-AR pathway. It can induce the activation of primordial follicles by inhibiting PTEN expression via the AR pathway [39]. The increase in AR expression can promote the proliferation of granulosa cells and inhibit apoptosis [40] and increase the content of insulin-like growth factor-1 (IGF-1) and growth differentiation factor-9 (GDF-9) in follicular fluid. It plays a role in follicle development and oocyte quality [41]. It can enhance the expression of follicle-stimulating hormone receptor (FSHR) in granulosa cells, improve the follicular sensitivity of FSH in the antral follicular phase, and increase the number of follicles [42]. Testosterone can improve the ovarian microenvironment and increase the expression of connexin 37 (Cx37) to promote ovarian response [43]. Low basal testosterone level is an important risk factor for low oocyte production after ovarian stimulation, which may affect the pregnancy rate of in vitro fertilization [44].

Luteal hormone is a hormone secreted by the ovary, which can inhibit the proliferation of granulosa cells induced by epidermal growth factor. It affects cell oxidative stress [45]. 8-Hydroxyguanosine (8-OHdG) and 4-hydroxynonenal (4-HNE) are the most common markers of oxidative stress. The increase in luteal hormone, 8-OHdG, and 4-HNE could cause the increase in oxidative stress response. Oxidative stress can cause enzyme inhibition, protein synthesis inhibition, DNA/RNA synthesis inhibition, and so on, which may be factors affecting oocyte quality [46]. Luteal hormone can also affect energy metabolism. Mitochondria are the factories of cell energy, which produce a lot of ATP to activate cell activity in aerobic respiration, and participate in steroid production and cell aging. Xu et al. found that the number of abnormal mitochondria of granulosa cells in patients with endometriosis was too large [47]. Cell aging is mainly due to the reduction of oxidative phosphorylation and ATP production [48]. Mitochondrial dysfunction can lead to spindle defects and chromosomal diseases of oocytes and affect the development, maturation, and fertilization of oocytes. The high level of luteal hormone and the decrease in the ATP level lead to the decrease in oocyte quality. The high level of luteinizing hormone will also cause the decrease in carnitine levels [31]. Carnitine can protect oocytes and improve the quality of oocytes. The increase in luteinizing hormone level will cause the decrease in oocyte quality and low fertilization rate. 17*β*-estradiol-3 and 17*β*-sulfate are the metabolites of estrogen. Estradiol is closely related to the fertilization ability of oocytes, and its metabolites could stimulate the production of progesterone. However, if the level of estradiol is too high, it can destroy the blood flow of uterus and ovary and may lead to anoxia of follicles to damage the development potential of embryos. Jiang et al. found that women with high estradiol levels had poor pregnancy outcomes [49]. In our study, there were significant differences in the number of oocytes obtained, the number of transplantable embryos, and IVF fertilization rate between the posttreatment group and the pretreatment group (*p* < 0.05).

Tryptophan may affect oocyte development and follicle quality through immunity. It can be transformed into melatonin. Melatonin can delay the aging of mouse oocytes after ovulation through SIRT1 MnSOD-dependent pathway [50], which can improve the inhibition of bisphenol A (BPA) on the meiosis and fertilization of oocytes to improve the quality of oocytes [51]. Tumor necrosis factor-*α* (TNF) is a kind of cytokine, which is produced not only by various cells in the immune system but also by various cells in the reproductive system. Indole is produced by the metabolism of tryptophan [52]. Indole may reduce TNF-mediated NF-KB activation. Oocytes, granulosa cells, and stromal cells are important sources of TNF, which can reduce the number of oocytes and primordial follicles by stimulating oocyte apoptosis. Therefore, TNF can be used as an important ovarian factor to determine the size of primordial follicle pool [53]. The absence of TNF receptors (tnfrsf1b) leads to the obvious acceleration of follicle growth, which is an important mediator of TNF function in the ovary and an important regulator of follicular development [54].

13,14-Dihydroretinol is related to retinoic acid metabolism. Retinoic acid is the intermediate metabolite of vitamin A. Some studies have found that vitamin A can protect ROS-induced cell oxidative stress [55]. 13,14-Dihydroretinol was upregulated after the intervention of LWDH pill to affect oocyte development and oocyte quality.

Acrylamide has toxic effects on the thyroid, red blood cells, skeletal muscle, smooth muscle, and nervous system. At the same time, it will affect the proliferation of mouse granulosa cells and the production of progesterone [56], resulting in the early apoptosis of oocytes [57]. *p*-Cresol sulfate is a biomarker of renal decline [58]. Some studies have shown that mitochondrial damage is one of the main pathological mechanisms of uremic poisoning [59]. Mitochondria, as the biological signal conduit of apoptosis, necrosis, and autophagy, affects oocyte quality and apoptosis. 5,8,11-Eicosapentaenoic is produced from arachidonic acid by cytochrome P450 (CYP) cyclooxygenase, which may be involved in lipid metabolism. The specific mechanism is unclear.

Metabolic changes are relevant to many diseases. In previous study, lipidomics has contributed an improved understanding of a potential underlying mechanism of ovarian aging and poor oocyte quality. Lipids seem to play a crucial role in ovary aging, and further analysis and validation of potential lipid biomarkers may facilitate improved IVF in the future [60]. In our study, a group of biomarkers were found and used to characterize disease based on metabolomics. It could be helpful to understand the key features of DOR and be useful for the prevention, diagnosis, and treatment of DOR.

## 5. Conclusions

A pseudotargeted metabolomics profiling on the follicular fluid was successfully established and integrally investigated. The follicular fluid metabolome was obviously altered in DOR patients after LWDH pill treatment. After the pseudotarget metabolomics screening, a total of 21 metabolic biomarker candidates, which may be related to DOR were characterized, and their correlative metabolomic pathways were revealed. In addition, it was speculated that the potential active targets of LWDH pill on DOR may be more comprehensive than chemical treatments. The study demonstrated that the metabolomics strategy might serve as a powerful approach for investigating the mechanisms of DOR and LWDH pills using the processing method, while providing solutions to explore the molecular basis of diseases and identify potential biomarkers.

## Figures and Tables

**Table 1:** Clinical background of subjects.

Item	Posttreatment	Pretreatment	Control	*p*
Age	34.75 ± 3.47	32.97 ± 4.01	31.14 ± 3.38	＞ 0.05
Infertility years	4.36 ± 2.16	3.66 ± 1.72	2.79 ± 1.62	＞ 0.05
BMI	22.17 ± 1.70	21.42 ± 1.74	21.63 ± 1.34	＞ 0.05
Basic LH	4.45 ± 1.62	4.69 ± 1.82	4.69 ± 1.78	＞ 0.05
Basic E2	48.43 ± 17.06	40.99 ± 20.43	38.45 ± 12.01	＞ 0.05
Basic P	0.62 ± 0.29	0.74 ± 0.24	0.66 ± 0.21	＞ 0.05

**Table 2:** Total integral of pretreatment and posttreatment using LWDH pill.

Item	Total integral	Coeffective	*T*	*P*
Pretreatment	15.11 ± 3.119	0.765	13.811	<0.001 ^*∗*^
Posttreatment	9.86 ± 2.445			

^∗^The difference between the two groups was of detectable statistical significance.

**Table 3:** The integral of each syndrome.

Item	Pretreatment	Posttreatment	Coeffective	*T*	*P*
YaoXiSuanRuan	3.21 ± 0.787	2.46 ± 1.427	0.403	3.000	0.006 ^*∗*^
WuXinFanRe	3.11 ± 0.567	1.29 ± 1.512	0.222	6.460	<0.001 ^*∗*^
KouGanYanZao	1.14 ± 0.705	0.64 ± 0.678	0.575	4.415	<0.001 ^*∗*^
Dizziness	1.18 ± 0.819	0.75 ± 0.441	0.231	2.714	0.011 ^*∗*^
Emaciation	0.32 ± 0.476	0.18 ± 0.39	0.677	2.121	0.043 ^*∗*^
Forgetfulness	0.43 ± 0.69	0.18 ± 0.39	0.668	2.553	0.017 ^*∗*^

^∗^The difference between the two groups was of detectable statistical significance.

**Table 4:** The number of follicles and endocrine parameters during the day of hCG.

Item	Posttreatment	Pretreatment	*P*
<14 mm follicles	3.75 ± 1.624	2.69 ± 1.775	0.022 ^*∗*^
≥14 mm follicles	4.04 ± 1.453	3.14 ± 1.552	0.028 ^*∗*^
LH	7.23 ± 4.16	5.2 ± 4.77	0.093
E2	1187.71 ± 712.681	1143.52 ± 700.787	0.814
P	1.17 ± 0.62	1.24 ± 0.66	0.681
Number of oocytes	3.75 ± 1.531	2.72 ± 1.412	0.011 ^*∗*^
Number of transplantable embryos	1.68 ± 1.124	0.97 ± 0.778	0.007 ^*∗*^
Number of high-quality embryos	0.32 ± 0.723	0.21 ± 0.412	0.464
Oocyte acquisition rate （%）	92.9%	86.8%	0.145
IVF fertilization rate （%）	73.9%	52.5%	0.007 ^*∗*^
Clinical pregnancy rate （%）	25%	20.7%	0.698

^∗^The difference between the two groups was of detectable statistical significance.

## Data Availability

The datasets analyzed during the current study are available from the corresponding author on reasonable request.
